# Positron emission tomography/computed tomography for bone tumors (Review)

**DOI:** 10.3892/ol.2014.2728

**Published:** 2014-11-21

**Authors:** AIKEREMUJIANG MUHEREMU, XIAOHUI NIU

**Affiliations:** Department of Orthopedic Oncology Surgery, Beijing JiShui Tan Hospital, Beijing 100035, P.R. China

**Keywords:** positron emission tomography/computed tomography, imaging, bone tumor

## Abstract

The aim of the present study was to investigate positron emission tomography (PET)/computed tomography (CT) and its applications for the diagnosis and treatment of bone tumors. The advantages and disadvantages of PET/CT were also evaluated and compared with other imaging methods and the prospects of PET/CT were discussed. The PubMed, Medline, Elsevier, Wanfang and China International Knowledge Infrastructure databases were searched for studies published between 1995 and 2013, using the terms ‘PET/CT’, ‘positron emission tomography’, ‘bone tumor’, ‘osteosarcoma’, ‘giant cell bone tumor’ and ‘Ewing sarcoma’. All the relevant information was extracted and analyzed. A total of 73 studies were selected for the final analysis. The extracted information indicated that at present, PET/CT is the imaging method that exhibits the highest sensitivity, specificity and accuracy. Although difficulties and problems remain to be solved, PET/CT is a promising non-invasive method for the diagnostic evaluation of and clinical guidance for bone tumors.

## 1. Introduction

Diagnostic imaging is of great importance for the initial staging and assessment of recurrence of various malignancies. The differentiation of benign and malignant intraosseous lesions may often be achieved using radiographical methods, including X-ray, computed tomography (CT) and magnetic resonance imaging (MRI) (1). Radiographs, including CT and X-ray, provide important information regarding the appearance, intraosseous extent and internal characteristics of bone tumors (1).

Positron emission tomography (PET) using 2-(fluorine-18)-2-deoxy-D-glucose (^18^F-FDG) has been used to differentiate malignant tumors from benign lesions (2). In the musculoskeletal system, findings of previous studies have shown a positive correlation between glucose consumption measured by ^18^F-FDG and the aggressiveness of musculoskeletal tumors (3). Griffeth *et al* (4) performed ^18^F-FDG PET studies on a series of 20 intraosseous lesions, where 14/15 malignant tumors and 4/5 benign lesions were correctly diagnosed, using a cut-off value of 2.0 for the maximum standardized uptake volume (SUV_max_). However, 13/15 of the malignant tumors were metastatic carcinomas. Aoki *et al* (5) identified a statistically significant difference between the SUV_max_ values of benign and malignant bone tumors in 52 bone lesions. However, the authors observed a significant overlap between the SUV_max_ values of a number of the benign and malignant bone tumors, and a high accumulation of ^18^F-FDG was reported in certain benign bone tumors. However, other studies have questioned the correlation between ^18^F-FDG accumulation and the malignant potential of bone tumors. Furthermore, the value of ^18^F-FDG in the musculoskeletal system has also been questioned. The intensity of ^18^F-fluorodeoxyglucose (FDG) uptake has been found to positively correlate with the histological grade of various malignancies. The combined metabolic and morphological information provided by the FDG PET/CT imaging has been shown to exhibit high sensitivity for initial staging and assessment of recurrence of soft tissue and osseous sarcomas in retrospective studies (6).

Dual-time point ^18^F-FDG PET is practical and may be useful for differentiating malignant tumors from benign ones (7). Serial FDG-PET imaging accurately classified histopathological response/non-response to neoadjuvant therapy in adult primary bone sarcomas. Notably, a combination of previously applied response criteria (reductions in SUV_max_ by ≥60% and a post-treatment tumor SUV_max_ of <2.5) provided the optimal response predictions (8).

## 2. Mechanisms of PET/CT

FDG PET and CT are imaging methods that have been found to be valuable in clinical application. Integrated PET/CT, which uses a combination of PET and CT in one imaging device, performs morphological and functional imaging in one procedure. Integrated PET/CT has been shown to be more accurate for lesion localization and characterization than PET and CT alone and the results obtained from PET and CT separately, following software-based fusion of the PET and CT data sets (16).

## 3. Current applications of PET/CT

### Diagnosis of bone tumor malignancy

^18^F-FDG PET enables the assessment of glucose metabolism and thus the metabolic activity of cancer tissue by calculating a SUV value (Table I). At present, ^18^F-FDG PET is successfully used and approved for procedure reimbursement in numerous cancer types, including lung cancer, melanoma, lymphoma, head and neck tumors, brain tumors, esophageal cancer and colorectal cancer (17).

### Osteosarcoma

Osteosarcoma is the most common primary malignancy of the bone, with the peak age of incidence ranging between 10 and 25 years (18). The initial stage and necrosis of tumors are the clearest prognostic factors (19). Osteosarcoma typically recurs within 2–3 years of surgery (20). Therefore, a longer follow-up period is required for a more complete assessment of survival. In a study by Cistaro *et al* (21), including 18 children with osteosarcoma and Ewing sarcoma, it was demonstrated that ^18^F-FDG PET/CT may be used to identify the metabolic characteristics of lung nodules in patients with bone sarcoma. In addition, a SUV_max_ (or SUV ratio of >1) was identified as a sensitive and specific marker to assess whether a lesion with a diameter of <6 mm is malignant or benign (22).

However, not all cancer types may be detected by PET/CT with high accuracy. Variable uptake is considered to be associated with the biological features of individual cancers, which has been observed in bronchoalveolar carcinomas, renal and thyroid cancers, several subtypes of malignant lymphoma and carcinoids, as well as the majority of prostate carcinomas. The prognostic relevance of this biological heterogeneity is not clear. However, in the majority of cases, FDG PET is a sensitive imaging modality for the detection, staging and re-staging, as well as the assessment of therapy response in cancers (23–25).

Findings of previous studies have shown that delayed PET (2–3 h following injection) may aid with the differentiation of malignant lesions from benign lesions (26,27). Sahlmann *et al* (28) investigated glucose metabolism in 17 patients with chronic bacterial osteomyelitis and four patients with malignant bone lesions using a dual time point ^18^F-FDG PET (30 and 90 min following injection). The authors hypothesized that dual time point ^18^F-FDG PET may be of value for the differentiation of chronic bacterial osteomyelitis and malignant bone lesions. However, the sample size of the study was small and only one type of benign bone lesion was investigated.

### Detection of cancer metastases

FDG PET has been shown to be useful for the detection of nodal and distant metastases in patients with soft tissue sarcomas when compared with that of conventional imaging (29). We hypothesized that PET/CT may also be an effective method for this purpose. In a study by Klaeser *et al* (30), 12 patients who were admitted to hospital for staging and restaging of breast cancer, non-small cell lung cancer, cervical cancer, soft tissue sarcoma and osteosarcoma were repositioned according to the results of PET-CT. Subsequently, intervention was planned according to a single-bed PET-CT acquisition of the affected region. The metabolically active sections of the lesions were accurately targeted in all the patients and representative samples were obtained in 92% of patients (30).

### Responses to therapeutic interventions

Changes in SUV following neoadjuvant chemotherapy (NACT) have been reported to be useful for the prediction of tumor response in osteosarcoma (31). The standard chemotherapy response evaluation used currently histologically assesses the tumor necrosis of the excised lesion (32), which has been reported to be the most important prognostic factor in osteosarcoma following NACT (33). However, as tumor necrosis may only be assessed in the resected specimens by surgery or biopsy, the continuation of ineffective chemotherapy may result in tumor resistance. ^18^F-FDG-PET-CT may serve as a surrogate for histological necrosis and may decrease the time required to evaluate the efficacy of NACT to determine the optimal therapeutic intervention (34).

The use of ^18^F-FDG PET was previously for the evaluation of osteosarcoma response to NACT (35,36). The association between tumor necrosis and outcome, as well as the association between worse survival and high metabolic activity in chemotherapy-naive tumors and high metabolic activity following chemotherapy, provide similar conclusions to those of previous studies, whereby ^18^F-FDG PET accurately reflects osteosarcoma response to chemotherapy. Despite the small number of mortalities and short follow-up period, these results are similar to those that indicate that survival is associated with SUV_max_ following chemotherapy (36).

The ^18^F-FDG uptake of the majority of pediatric sarcomas is high, indicating that ^18^F-FDG PET/CT imaging may be used to monitor responses to therapeutic interventions (37).

### Prediction of prognosis

Costelloe *et al* (38) analyzed the SUV_max_ of 33 patients with osteosarcoma using ^18^F-FDG positron-emission tomography-computed tomography (PET/CT) and found that although the location of high SUV_max_ values was not associated with the five-year survival rate of patients, a high SUV_max_ value was significantly associated with a low five-year survival rate of patients (38).

## 4. Comparison of PET/CT and other imaging modalities

### Bone scans

Although few studies have compared the diagnostic accuracy of ^18^F-FDG PET with that of bone scans, ^18^F-FDG PET has demonstrated a higher sensitivity than that of bone scans for the detection of bone or bone marrow metastases. The detection rates of ^18^F-FDG PET in previous studies were 80–100% (39,40), while the detection rates of bone scans were 61 and 75% (39).

### MRI

MRI is an extremely sensitive method for the detection of bone marrow abnormalities, cortical destruction or soft tissue tumors adjacent to or infiltrating neighboring bone (40). However, the morphological appearance of numerous lesions is non-specific. With certain entities, the diagnosis may be difficult with radiographical imaging or MRI.

### PET and CT

Antoch *et al* (41) compared the value of PET, CT and dual-modality PET/CT imaging for assessing gastro-intestinal stromal tumor (GIST) response to imatinib therapy and demonstrated that image fusion with combined PET/CT may provide additional information in individual cases when compared with side-by-side PET and CT (41).

Although tumor size and infiltration of adjacent structures may be accurately assessed using CT (42), PET has been demonstrated to be markedly more sensitive and specific in the detection and characterization of metastases to mediastinal lymph nodes (43). The limited anatomical information yielded by PET may be overcome by combining the functional PET data with morphological CT data.

In a study by Yi *et al* (44), which included 119 patients, the sensitivity and accuracy of PET/CT for the accuracy for pulmonary nodule characterization was investigated and was found to be significantly higher than that of helical dynamic CT.

Regarding the combination of PET/CT and conventional imaging, the accuracy of T staging for osteosarcoma was significantly more accurate than PET alone. However no significant differences were identified between conventional methods or PET/CT. PET/CT is significantly more accurate for the N and M staging of osteosarcoma than conventional imaging methods (P<0.01). These results indicated that the combination of PET/CT and conventional imaging is the optimal preoperative staging method for bone and soft tissue sarcomas, due to its significantly higher diagnostic accuracy (45).

## 5. Problems and prospects for PET/CT

Although PET/CT has been found to be useful in the diagnosis and treatment of musculoskeletal tumors, a number of issues remain that require further study. Although these issues remain a challenge, they may present potential opportunities for the wide application of PET/CT in orthopedic oncology.

### Application of SUV

In two previous studies, SUV_max_ was used as a parameter for response prediction (46,47). In these studies, SUV2/SUV1 and SUV2 were found to correlate with histological response. In addition, an SUV2 of <2 and an SUV2/SUV1 of <0.4 were accurate response indicators.

### Application of tumor volume measurements

Tumor volume measurements are easier to obtain and more reproducible in osteosarcoma than in other tumor types, when an appropriate MRI technique is used (48).

### Combined PET/CT and MRI systems

Combined PET/MRI systems allow the simultaneous or sequential acquisition of PET and MRI images (49), and literature regarding previous studies and the initial clinical experiences with PET/MRI in oncology are available (50).

Combined metabolic and volumetric information on ^18^F-FDG PET and MRI scans, obtained prior to and following chemotherapy completion, may be used to predict the histological response to NACT in osteosarcoma (51).

For specific tumors that are not PET/CT sensitive, the combined use of PET/CT and MRI may significantly increase the sensitivity of diagnosis (52).

Accurate anatomic localization of functional abnormalities observed with PET is known to be difficult. Although non-specific tracers, such as ^18^F-FDG, visualize certain normal anatomical structures, the spatial resolution is generally inadequate for the localization of pathology. Combining PET with a high-resolution anatomical imaging modality, such as CT, may resolve this localization issue. However, the images captured from the two modalities must be accurately coregistered.

## 6. Conclusion

The combined metabolic and morphological information provided by FDG PET/CT imaging provides sensitivity for initial staging and the assessment of recurrence of soft tissue and osseous sarcomas.

## Figures and Tables

**Table I:** Summary of previous studies using positron emission tomography/computed tomography for the diagnosis of musculoskeletal tumors.

Study, year (Refs.)	No. of patients	SUV_max_		SUV_max_ cut off	Sensitivity, %	Specificity, %	Accuracy, %

		Benign	Malignant				
Fuglø 2012 (9)	89				95.8	92.7	93.1
Aoki 2003 (5)	114	1.80 (SD, ±1.42)	4.20 (SD, ±3.16)				
Shin 2008 (10)	34	2.9 (range, 0.6–5.5)	12.0 (range, 4.3–45.7)	4.7	89.6	86.7	88.2
Piperkova 2008 (11)	93				100	95.9	
Charest 2009 (6)	212					93.9	
Iagaru 2006 (12)	44				100	93.3	
Strobel (13)	50	3.5 (range, 1.6–8.0)	3.5 ( 1.6–8.0) (range, 1.6–8.0)		91	77	86
Shin 2008 (14)	87	5.1	8.8	4.7	80	63	70
Eary 2002 (15)	209		6 (range, 3.8–11.3)				

SD, standard deviation; SUV_max_, maximum standardized uptake volume.
